# Chinese pre-service general education teachers’ attitudes and self-efficacy toward inclusive education

**DOI:** 10.3389/fpsyg.2025.1490144

**Published:** 2025-09-09

**Authors:** Yi Yang, Hsi-Wu Liu, Chao-An Chi, Moon Young Savana Bak

**Affiliations:** ^1^Department of Special Education, Lingnan Normal University, Lingnan Normal University, Zhanjiang, China; ^2^Department of Educational Psychology, University of Minnesota, Twin Cities, St. Paul, MN, United States

**Keywords:** inclusive education, teacher attitude, self-efficacy, teacher education, students with disabilities

## Abstract

In China, full inclusion of students with disabilities in general education classrooms with their same-aged peers is strongly encouraged but not yet legally mandated. As such, the attitudes of general education teachers to include students with disabilities in their classrooms and teacher self-efficacy of inclusive education are two important factors that can decide the success or failure of inclusion. The current study conducted a survey to understand pre-service general education teachers’ attitudes and self-efficacy of inclusive education. We surveyed 587 pre-service general education teachers in the Guangdong province of China using the Sentiment, Attitudes, and Concerns about Inclusive Education-Revised Scale to measure teacher perspectives and the Teacher Efficacy for Inclusive Practices Scale to measure teacher efficacy. The results of the showed upper-middle level scores for both attitudes (3.15 points of 5) and efficacy (3.17 points of 5) in participating pre-service teachers with a statistically significant positive correlation between attitude and efficacy. Implications and future directions for providing adequate training to pre-service general educators to promote inclusive education for students with disabilities are also discussed.

## Introduction

In China, the inclusion of students with disabilities in general education classrooms began in the 1980s (Yan et al., 2021). Students with disabilities in China are placed in three different settings—a special education school, a self-contained special education classroom within a general education school, and full inclusion in general education classrooms—based on the level of support students need for academic learning. The decision is made based on the resources available in each school and the recommendations of the administrators and teachers at the school (Chan et al., 2023). However, it was not until 2014 that the Ministry of Education officially elevated inclusive education as a key objective for special education development, making it the recommended practice for students with disabilities. Since then, the number of students with disabilities in general education schools has increased from 270,800 in 2017 to 304,300 in 2018, an increase of nearly 13% (Ministry of Education of the People’s Republic of China, 2017). To improve the quality of inclusive education, the State Council and the Ministry of Education (State Council of the People’s Republic of China, 2017; State Council of the People’s Republic of China, 2020) introduced measures requiring schools to provide not only access to mainstream education but also high-quality inclusive education for students with disabilities. Some of these measures included the development and piloting of special education related courses for pre-service general education teachers in some universities, a special education course requirement for master’s degree in education, and the inclusion of special education related questions in teacher licensing examinations. While enrollment of students with disabilities in general education schools is increasing, their actual inclusion varies significantly (Qu, 2022). The literature emphasizes the need for meaningful inclusion, ensuring that students with disabilities participate fully in school life alongside their peers (Downing and Peckham-Hardin, 2007; Skinner et al., 2022). Effective systems-level support is essential for successful inclusive education (Rapp and Corral-Granados, 2024). However, general education teachers are crucial for implementing quality inclusive practices in the classroom (Hernandez et al., 2016).

Effective inclusive education requires teachers to respect each student’s diverse skills and needs and provide behavioral, emotional, and social support to promote true inclusion in the classroom. Therefore, general education teachers must be skilled in instructional techniques, including universal design for learning, adapting strategies for diverse learners, and applying fair assessments (Turnbull et al., 2020). For students with disabilities, teachers need to develop individualized educational plans, adjust curricula, and implement tailored evaluations based on each student’s learning needs. Ultimately, the responsibility for effective inclusive education lies with the classroom teacher, who must integrate grade-level objectives with individualized practices (Mulholland and O’Connor, 2016; Solis et al., 2012).

One important factor influencing the quality of inclusive education is general education teachers’ attitude toward inclusive education (Dignath et al., 2022; Leyser et al., 2011). In psychology, attitude refers to the degree of favorability toward a certain target encompassing objects, individuals, environments, or abstract ideas (Eagly, 1993). A teacher’s attitude can indicate their preconceived notions about practices like inclusive education and affect their pedagogical behaviors (Saloviita, 2020). Thus, these attitudes are often rooted in the teachers’ value systems (Savolainen et al., 2012). Research indicates that general education teachers with positive attitudes toward inclusive education are more proactive in raising awareness about disabilities and fostering meaningful interactions between students with and without disabilities (Saloviita, 2020; Yu et al., 2015). Additionally, such teachers are more likely to create suitable learning opportunities for students with disabilities (Cameron and Cook, 2013). In contrast, teachers with negative attitudes often struggle with self-efficacy in teaching these students (Yada et al., 2022). Furthermore, studies suggest that a teacher’s attitude can influence teaching efficacy, with positive attitudes leading to better student outcomes compared to negative ones (Denessen et al., 2022).

Teacher self-efficacy refers to a teacher’s belief in their ability to positively influence students’ academic performance (Lazarides and Warner, 2020). It is a crucial factor in explaining differences in teaching effectiveness (Poulou et al., 2018). Research shows that teachers with high self-efficacy actively seek solutions for students’ learning difficulties and take responsibility for teaching quality (Zee and Koomen, 2016). In contrast, teachers with low self-efficacy may struggle to address classroom problems and often attribute their challenges to external factors beyond their control, such as a student’s learning abilities or systemic issues (Viel-Ruma et al., 2010; Zee and Koomen, 2016). Consequently, a general education teacher’s self-efficacy regarding inclusive education can also influence their attitudes toward it (Savolainen et al., 2012; Wray et al., 2022). Many studies have explored the relationship between attitudes toward inclusive education and teacher self-efficacy, most finding a positive correlation (see Wray et al., 2022). For instance, Yada and Savolainen (2017) reported that general education teachers’ efficacy in behavioral management and collaboration significantly affects their attitudes toward inclusive education. Teachers who feel competent in managing inclusive education tend to have more positive attitudes and a higher sense of efficacy, leading to better quality inclusion.

While disability awareness and societal attitudes toward inclusion are improving in China, there is still a significant shortage of special educators graduating from dedicated programs (Feng, 2014). Many pre-service and in-service general education teachers may lack sufficient training in special education (e.g., individualized education) and inclusive practices (e.g., universal design for learning). Malinen et al. (2012) studied the self-efficacy of in-service teachers in Beijing and found that the efficacy of cooperation is the most important predictor of attitudes toward inclusive education. Given the current shortage of special educators and the push for inclusive education for students with disabilities, the literature on teacher attitudes regarding inclusive education in China primarily focuses on in-service teachers (e.g., Hu et al., 2019, and Jiang and Huang, 2019 for kindergarten teachers; Gao, 2020, and Yang and Jia, 2019 for elementary teachers) and comparisons between the attitudes of teachers with different responsibilities—such as rural versus urban and special education versus mainstream—toward inclusion (e.g., Zan et al., 2011).

However, there are relatively few studies focusing on pre-service general education teachers and their attitudes and perceived self-efficacy regarding inclusive education. In Wang and Zhao’s (2019) survey of 370 pre-service preschool education students in their first to third year of college training, participants were generally favorable toward the importance of education for students with disabilities, however the majority of the participants (i.e., 67.4%) believed that students with disabilities should receive education in a special education setting. In Yang and Jia’s (2019) used a survey and interview method to investigate the attitudes toward inclusive education in 234 pre-service elementary education students. The participants showed overall positive attitudes toward inclusive education for students with disabilities, however, this positive attitude declined for the participants who were in their third and fourth years of college. Yang and Jia’s (2019) study did not find any statistically significant difference regarding the participants’ gender, experience working with individuals with disabilities, and previous training in special education. More recently, Tian (2020) surveyed 800 pre-service general education students and found that the attitudes toward inclusive education were less positive in fourth year students compared to students in their first to third years of training. Although some research has examined the relationship between teachers’ backgrounds (e.g., gender, age, job responsibilities, and interactions with individuals with disabilities) and their attitudes toward inclusive education (e.g., Tian, 2020; Wang and Zhao, 2019), the results are inconclusive regarding whether key background variables, such as completing special education courses or receiving specific training in inclusive education, positively affect pre-service general education teachers’ attitudes and self-efficacy. These future educators will be responsible for providing appropriate education and support for students with disabilities in inclusive settings (i.e., their classrooms). The quality of education and training for pre-service general education teachers directly impacts the inclusive education of students with disabilities, and their attitudes and self-efficacy will inevitably influence the educational quality for these students.

To address this gap in literature, the current study aimed to explore the attitudes toward inclusive education in pre-service general education teachers, their levels of self-efficacy, and the relationship, if any, between attitudes and self-efficacy, and any teacher-level characteristics (i.e., demographic variables).

Specifically, we ask the following research questions:

1. What are the attitudes and self-efficacy of pre-service general education teachers toward inclusive education?
2. Is there a correlation between the attitudes and self-efficacy of pre-service general education teachers toward inclusive education?
3. Are there any differences between attitudes and self-efficacy of pre-service general education teachers when considering demographic variables?

## Method

This study was approved by the Institutional Review Board of the first author’s institution.

### Participants and recruitment

Participants were recruited from all four colleges of education in Guangdong Province, China. The first author sent letters and emails to the colleges and faculty in general education teacher preparation programs, introducing the study’s objective and asking them to share it with interested pre-service general education students in mid-September of 2021. The solicitation email included a QR code link to an online survey (Wenjuanxing, a Chinese survey application) for participation. The survey was open for one month (i.e., September 21 to October 21, 2021). At the end of the survey period, we collected 601 responses, of which 587 were valid (i.e., participants were pre-service general education teachers) and completed. Detailed demographics, including sex, year of study (e.g., freshman, sophomore), and experience with special education, are provided in Table 1.

**Table 1:** Detailed demographics of participants.

		*n* (*N* = 587)	Percentage
Gender	Male	94	16.0%
	Female	493	84.0%
Year of study	Year 1	46	7.8%
	Year 2	59	10.1%
	Year 3	362	61.7%
	Year 4	120	20.4%
Enrolled in special education courses in college	Yes	151	25.7%
	No	436	74.3%
Attended K-12 class/school with peers with disabilities	Yes	204	34.8%
	No	383	65.2%
Has family members with disabilities	Yes	21	3.6%
	No	566	96.4%
Has participated in extra-curricular activities with/for individuals with disabilities	Yes	120	20.4%
	No	467	79.6%

### Measures

#### Sentiment, Attitudes, and Concerns about Inclusive Education-Revised (SACIE-R)

We used the Sentiment, Attitudes, and Concerns about Inclusive Education-Revised (SACIE-R) scale (Forlin et al., 2011) to assess the attitudes of pre-service general education teachers toward inclusive education. Based on SACIE (Loreman et al., 2007), this scale has been modified and supplemented by Forlin et al. (2011) for use in various countries. The SACIE-R scale is divided into three dimensions—Sentiments, Attitudes, and Concerns—and contains a total of fifteen items. Items in Sentiments pertain to the degree of comfort a teacher may feel while interacting with students with disabilities. Items in Attitude pertain to the degree of a teacher’s acceptance of specialized needs for students with disabilities in education. Items in Concerns pertain to potential worries a teacher may feel when they practice inclusive education. Although the dimension of Attitude is separately represented within the SACIE-R, all three dimensions—Sentiments, Attitudes, and Concerns—are critical to understanding the broad construct of a teacher’s attitude toward inclusive education as they are interconnected and represent the degree to which a teacher may believe in and show support for inclusive education (Forlin et al., 2011). The SACIE-R scale uses a 4-point rating scale (i.e., 1 = strongly disagree, 2 = disagree, 3 = agree, and 4 = strongly agree) and dimensions of Sentiments and Concerns are scored inversely. The higher the total score, the more positive the attitude toward inclusive education.

#### Teacher Efficacy for Inclusive Practices Scale (TEIP)

We used the Teacher Efficacy for Inclusive Practices (TEIP; Sharma et al., 2012) scale to evaluate self-efficacy of pre-service general education teachers regarding inclusive education. The TEIP is divided into three dimensions—Inclusive Instruction, Collaboration, and Managing Behavior—and was designed to measure teachers’ confidence in their ability to teach in an inclusive education environment. The Inclusive Instruction dimension includes six items and measures teacher efficacy in providing effective instruction for diverse learners in their classroom. The Collaboration dimension includes six items and measures teacher efficacy in collaborating with caregivers and other professionals to support students with disabilities in their classrooms. The Managing Behavior dimension includes six items and measures teacher efficacy in preventing or providing positive solutions for disruptive behavior. The TEIP scale uses a 6-point Likert (1 = strongly disagree to 6 = strongly agree) measurement with no inverse scoring. A higher total score indicates a higher sense of efficacy when implementing inclusive education.

### Procedures

#### Translation and adaptations of the scales

Both scales were translated from English to Chinese by the authors. After translation, we recruited two external auditors—an English professor from a Chinese university fluent in both languages and a Chinese student in a special education PhD program at a U.S. university—to proofread and revise any discrepancies. The translated scales were then evaluated by three external professionals in special education (one university professor and two teachers from a special education school) to ensure that the questions were clear, and the translations were valid. Based on their feedback, we adopted a unified five-point scoring system for both scales to avoid forced responses that can occur with four or six-point scales and to minimize confusion for participants when completing two separate scales.

Exploratory factor analysis was conducted to confirm the structural validity and reliability of the adapted scales on 148 college students majoring in general education prior to the current study. These students were not participants in the current study. A low factor loading was evident in two items within the Sentiments dimension of SACIE-R (i.e., ‘I am afraid that one day I will become an individual with a disability’ and ‘I will be afraid should I become disabled’). Similarly, a low factor loading was present in the Inclusive Instruction dimension (i.e., ‘I believe I am capable of making arrangements for students to conduct peer activities or group learning activities’), the Collaboration dimension (i.e., ‘I can assist students’ families to guide children’s adaptation to school life’), and the Managing Behavior dimension (‘I believe I am capable of preventing problematic behaviors in class before they appear’) of TEIP. The items with low factor loading were deleted from respective scales. The Cronbach *α* values of both scales were adequate and the final scale items, loading, and reliability for both scales and the Cronbach α values are presented in the Supplementary materials.

#### Data analysis

The results of the SACIE-R scale and the TEIP scale were tabulated using descriptive analysis via IBM SPSS 23 to answer the first research question: what are the attitudes and self-efficacy of pre-service general education teachers toward inclusive education? We conducted a Pearson correlational analysis using SPSS to answer the second research question: is there a correlation between the attitudes and self-efficacy of pre-service general education teachers toward inclusive education? We also planned to conduct further analysis (i.e., multiple linear regression) based on the results of the correlational analysis to investigate the strength of the correlation between the two scales and their dimensions. Finally, we conducted a *t*-test and a one-way ANOVA to answer the third research question: are there any differences between attitudes and self-efficacy of pre-service general education teachers when considering demographic variables? All assumptions were checked and met before analysis.

## Results

### Descriptive results

We used descriptive statistics to investigate the attitudes and self-efficacy of pre-service general education teachers toward inclusive education and answer the first research question.

The results of the SACIE-R scale showed that the attitude of pre-service general education teachers toward inclusive education is situated above the medium level (*M* = 3.15, *SD* = 0.47). By dimensions, Attitudes (*M* = 3.28, *SD* = 0.88) had the highest score, followed by Sentiments (*M* = 3.21, *SD* = 0.85) and Concerns (*M* = 2.98, *SD* = 0.75). Detailed results are presented in Table 2.

**Table 2:** Results of the Sentiment, Attitudes, and Concerns about Inclusive Education-Revised Scale.

	*M* (SD)
Total score	3.15 (0.47)
Sentiments	3.21 (0.85)
Attitudes	3.28 (0.88)
Concerns	2.98 (0.75)

The results of the TEIP scale showed that self-efficacy of inclusive education in participating pre-service general education teachers was above the medium level (*M* = 3.18, *SD* = 0.59). By dimensions, Collaboration (*M* = 3.21, *SD* = 0.69) had the highest score, followed by Inclusive Instruction (*M* = 3.18, *SD* = 0.64), and Managing Behavior (*M* = 3.14, *SD* = 0.59). Detailed results are presented in Table 3.

**Table 3:** Results of the Teacher Efficacy for Inclusive Practices Scale.

	*M* (SD)
Total score	3.18 (0.59)
Inclusive instruction	3.18 (0.64)
Collaboration	3.21 (0.69)
Managing behavior	3.14 (0.59)

### Correlation between attitudes and self-efficacy

A Pearson correlational analysis was conducted to determine the correlation between the SCAIE-R and TEIP scales and answer the second research question (see Cicchetti, 1994 for interpretation of coefficients). The analysis revealed a significant correlation between the total scores of the two scales (*r* = 0.39, *p* < 0.01). Within various dimensions, there was a moderately positive correlation, with the strongest correlation found between the Acceptance dimension of the SCAIE-R and the Collaboration dimension of the TEIP (*r* = 0.52, *p* < 0.01), followed by the Inclusive Instruction dimension (*r* = 0.48, *p* < 0.01) and the Managing Behavior dimension (*r* = 0.35, *p* < 0.01). Additionally, the Concerns dimensions were significantly negatively correlated with the Inclusive Instruction and Collaboration dimensions of the TEIP. Detailed correlation results are presented in Table 4.

**Table 4:** Correlational analysis results between the SCAIE-R Scale and the TEIP Scale.

	SCAIE-R total score	Sentiments	Attitudes	Concerns
TEIP total score	0.39^**^	0.37^**^	0.50^**^	−0.19^**^
Inclusive instruction	0.37^**^	0.34^**^	0.48^**^	−0.20^**^
Collaboration	0.35^**^	0.35^**^	0.52^**^	−0.28^**^
Managing behavior	0.36^**^	0.30^**^	0.35^**^	0.03

SCAIE-R, Sentiment, Attitudes, and Concerns about Inclusive Education-Revised; TEIP, Teacher Efficacy for Inclusive Practices. **p < 0.01.

We employed a stepwise multiple linear regression analysis to explore the relationship between the SCAIE-R and the three dimensions of the TEIP (Inclusive Instruction, Collaboration, and Managing Behavior). The results indicated a statistically significant relationship (*F* = 32.83, *p* < 0.001), with the Inclusive Instruction and Managing Behavior dimensions explaining 15% of the SCAIE-R results (*R^2^* = 0.15). The *β* value for both dimensions was 0.16 (*p* < 0.001). Notably, the relationship with Inclusive Instruction (*β* = 0.22, *p* < 0.001) was slightly stronger than that with Managing Behavior (*β* = 0.20, *p* < 0.001). Detailed regression results are presented in Table 5.

**Table 5:** Multiple linear regression between the SCAIE-R and TEIP Scale.

Variable	Non-standardized coefficient		Standardized coefficient *β*	*t*(*p*)	*TOL*	*VIF*
	*B*	*SE*				
(Constant)	2.12	0.10		21.81		
Inclusive Instruction	0.16	0.04	0.22	4.20^***^	0.51	1.95
Managing Behavior	0.16	0.04	0.20	3.82^***^	0.51	1.95
*F(p)*	32.83^***^					
*R* ^2^	0.15					
Durbin-Watson	1.86					

SCAIE-R, Sentiment, Attitudes, and Concerns about Inclusive Education-Revised; TEIP, Teacher Efficacy for Inclusive Practices. ***p < 0.001.

### Demographic variables and scale scores

We conducted a *t*-test and a one-way ANOVA to identify any differences between attitudes and self-efficacy of pre-service general education teachers and teacher-level demographic variables to answer the third research question. Specifically, we compared the results of the overall SCAIE-R scale and also by each dimension of the SCAIE-R scale.

The results of the *t*-test revealed a significant difference between male and female participants in the SCAIE-R scale (*t* = 2.20, *p* < 0.05). By dimension, there was a significant difference between male and female participants in the Sentiment dimension (*t* = 2.01, *p* < 0.05) and the Attitudes dimension (*t* = 2.01, *p* < 0.05) of the SCAIE-R scale.

Concerning years of study (e.g., freshman, sophomore) of the participants, there were significant differences in the total scores and the three dimensions of the SCAIE-R scale. The third- and fourth-year participants scored higher than the first and second years in the Sentiments dimension (*F* = 41.10, *p* < 0.001) and the Acceptance dimension (*F* = 46.37, *p* < 0.001) of the SCAIE-R scale. For the Concerns dimension of the SCAIE-R scale, the first- and second-year participants scored higher than the third- and fourth-year participants (*F* = 15.78, *p* < 0.001).

Regarding experience with special education courses, participants who had enrolled in special education courses scored significantly higher than those who had not enrolled in any special courses in the SCAIE-R scale (*t* = −3.92, *p* < 0.01) and particularly in the Sentiments dimension (*t* = −2.16, *p* < 0.05).

Participants who had family members with disabilities or attended school with peers with disabilities had a significant difference from participant who did not only in the Attitudes dimension on the SCAIE-R scale (*t* = 1.98, *p* < 0.05). Detailed results of the differences between the SCAIE-R scale and teacher-level demographic variables are presented in Table 6.

**Table 6:** Comparisons between demographic variables and the SCAIE-R Scale.

Demographic variables (*n*)		Total score	Sentiments	Attitudes	Concerns
		*M* (SD)	*M* (SD)	*M* (SD)	*M* (SD)
Gender	Male (*n* = 94)	3.24 (0.43)	3.37 (0.77)	3.49 (0.79)	2.91 (0.73)
	Female (*n* = 493)	3.13 (0.48)	3.18 (0.86)	3.23 (0.89)	2.99 (0.75)
	*p*	0.029	0.047	0.005	0.321
	*t*	2.20*	2.01*	2.83**	−0.996
Year of study	Year 1 (*n* = 46)	2.76 (0.45)	2.57 (0.73)	2.33 (0.88)	3.31 (0.61)
	Year 2 (*n* = 59)	2.90 (0.43)	2.47 (0.86)	2.62 (0.76)	3.45 (0.64)
	Year 3 (*n* = 362)	3.27 (0.47)	3.45 (0.78)	3.51 (0.76)	2.93 (0.67)
	Year 4 (*n* = 120)	3.03 (0.32)	3.22 (0.85)	3.24 (0.86)	2.77 (0.92)
	*p*	0.000	0.000	0.000	0.000
	*F*	30.28***	41.10***	46.37***	15.78***
Special education course enrolment	Yes (*n* = 151)	3.18 (0.35)	3.26 (0.73)	3.29 (0.90)	3.03 (0.87)
	No (*n* = 436)	3.04 (0.50)	3.10 (0.88)	3.22 (0.88)	2.82 (0.69)
	*p*	0.00	0.03	0.38	0.07
	*t*	−3.92**	−2.16*	−0.87	−2.73
Peers with disabilities in K-12 school	Yes (*n* = 204)	3.12 (0.44)	3.15 (0.83)	3.25 (0.93)	2.97 (0.74)
	No (*n* = 383)	3.17 (0.48)	3.25 (0.93)	3.29 (0.85)	2.98 (0.75)
	*p*	0.30	0.15	0.64	0.86
	*t*	−1.04	−1.44	−0.43	−0.17
Family members with disabilities	Yes (*n* = 21)	3.22 (0.43)	3.28 (0.74)	3.65 (1.10)	2.77 (0.82)
	No (*n* = 566)	3.14 (0.47)	3.21 (0.85)	3.26 (0.87)	3.00 (0.75)
	*p*	0.43	0.70	0.048	0.20
	*t*	0.80	0.39	1.98*	−1.29
Extra-curricular activities with individuals for/with disabilities	Yes (*n* = 120)	3.19 (0.49)	3.28 (0.89)	3.30 (0.88)	3.02 (0.74)
	No (*n* = 467)	3.14 (0.47)	3.20 (0.83)	3.27 (0.88)	2.97 (0.75)
	*p*	0.30	0.35	0.78	0.46
	*t*	1.05	0.94	0.28	0.75

SCAIE-R, Sentiment, Attitudes, and Concerns about Inclusive Education-Revised. ^*^p < 0.05, ^**^p < 0.01, ^***^p < 0.001.

We employed a post-hoc multiple linear regression analysis to further examine the relationship between participants’ demographic characteristics and their results on the TEIP scale for the demographic characteristics that showed statistically significant results with the SCAIE-R scale. The results of the multiple linear regression model revealed a statistically significant relationship between the demographic characteristics of the participants and the TEIP scale results (*F* = 72.32, *p* < 0.001). Specifically, the variables of year of study and enrollment in special education courses accounted for 20% of the variance in the TEIP results (*R*^2^ = 0.20). The *β* values for year of study and enrollment in special education courses were 0.53 (*p* < 0.001) and 0.39 (*p* < 0.001), respectively, both of which were significant. The results of the multiple linear regression between the demographics of the participants and the TEIP scale are presented in Table 7.

**Table 7:** Multiple linear regression between the demographics of participants and TEIP Scale.

Variable	Non-standardized coefficient		Standardized coefficient β	*t*(*p*)	*TOL*	*VIF*
	*B*	*SE*				
(Constant)	27.30	2.28		11.98		
Year of study	6.85	0.58	0.53	12.00^***^	0.67	1.48
Enrolled in special education courses in college	9.04	1.04	0.39	8.67^***^	0.67	1.48
*F(p)*	72.32^***^					
*R* ^2^	0.20					
Durbin-Watson	1.72					

TEIP, Teacher Efficacy for Inclusive Practices. ***p < 0.001.

There were no significant differences between participants who had extra-curricular experience with or for individuals with disabilities and participants who did not on both the SCAIE-R scale and the TEIP scale in all dimensions.

## Discussion

The current study examined correlations between attitudes and teacher self-efficacy toward inclusive education in pre-service general education teachers from four universities in Guangdong Province, China. Descriptive results indicated that participants’ attitudes, measured by the SCAIE-R scale, and teacher self-efficacy, measured by the TEIP scale, were at an upper-middle level. This aligns with previous research on in-service teachers’ attitudes (e.g., Yang and Jia, 2019; Chen and Chen, 2017) and self-efficacy (e.g., Wu and Luo, 2020; Xiong et al., 2019; Zan et al., 2011) in China. Among the three dimensions of the SCAIE-R scale, the highest scores were in the Acceptance dimension, followed by Sentiments, while the Concerns dimension received the lowest scores. Moreover, teacher self-efficacy, as measured by the TEIP scale, was significantly negatively correlated with the Concerns dimension of the SCAIE-R scale. This means that participants with higher self-efficacy reported more positive attitudes toward inclusive education.

Our study found that inclusive education attitudes as measured by the SCAIE-R scale were significantly different regarding the three demographic variables of gender, years of study, and special education learning experience. Regarding gender, the total score of male students’ attitudes toward inclusive education was significantly higher than that of females (*t* = 2.20, *p* < 0.05). Previous studies have shown mixed results regarding gender and attitudes toward inclusive education (e.g., Li, 2016; Zhao and Li, 2015). One reason for the inconsistent results in extant literature may be due to the different inclusive education attitudes scales used in each study. Another reason could be that in most studies there were more females than male participants. For example, Yang and Jia (2019) found no significant difference in the inclusive education attitudes in 188 female participants compared to 46 male participants.

Concerning years of study, there are significant differences in the total scores for the SCAIE-R scale and the scores of the three sub-dimensions. In this study, third-year students had significantly higher scores on the SCAIE-R scale, particularly in the Sentiments and Attitudes dimensions, compared to first- and second-year students. Previous research has shown that attitudes and self-efficacy toward inclusive education can improve with increased professional knowledge, teaching skills, and practical experience (Leyser et al., 2011; Yada et al., 2022). Another reason for the higher SCAIE-R scores among senior students may be that most of the four universities offer one to two special education courses for third-year students and above. Our study also found significant differences in the total scores of the SCAIE-R scale and the sub-dimension of *Sentiments* in terms of whether a participant had a prior learning experience related to special education. Acquiring special education knowledge helps pre-service general education teachers understand the strengths and needs of students with disabilities and practice teaching methods that facilitate their learning.

However, it is noteworthy that in the Concerns dimension of the SCAIE-R scale, third- and fourth-year participants scored significantly higher than first- and second-year participants. This suggests that senior students may have more concerns about the practical aspects of inclusive education as they prepare for teaching. These results of the current study are consistent with Yang and Jia’s (2019) study and Tian’s (2020) study that showed students who were in their third and fourth year of study displayed less positive attitude toward inclusive education. Research shows that while most in-service general education teachers believe in the importance of inclusive education and hold positive attitudes, they still have concerns about its implementation (e.g., Frankel et al., 2014; Nguyen and Hughes, 2012). Previous studies have also shown that although pre-service general education students believed in the importance of education for students with disabilities, they also believed that a special education environment would be more appropriate for students with disabilities (Wang and Zhao, 2019). One reason for this may be the limited hands-on and field practice in effective inclusive education methods and classroom management skills, as special education courses for general education pre-service teachers often cover only basic topics like the history of special education, referral processes, laws, and a summary of disabilities (Dignath et al., 2022; Wray et al., 2022).

One possible explanation for participants’ moderate level of concern toward inclusive education could be their proactive acceptance of the academic needs of students with disabilities. However, most participants had not taken a course in special education or inclusive education. Demographic information suggests that relatively few pre-service general education teachers in China receive training for teaching students with disabilities. The importance of training pre-service teachers helps explain the differences in SCAIE-R and TEIP scale scores by year of study (e.g., freshmen, seniors). A closer examination of year of study (*β* = 0.53, *p* < 0.001) on the TEIP scale results were slightly stronger than the effect of enrollment in special education courses (*β* = 0.39, *p* < 0.001). The combination of increased years of study and taking special education courses may significantly enhance teachers’ efficacy for inclusive practices. Consequently, insufficient training may affect in-service teachers’ attitudes and the implementation of quality inclusive education. Given this lack of foundational knowledge, it is understandable that pre-service teachers may feel uncertain about their future practices in inclusive classrooms. Previous studies have shown that many in-service general education teachers express concerns about their qualifications to provide effective inclusive education due to inadequate special education training during their preparation (e.g., Crispel and Kasperski, 2021; Kurth and Foley, 2014).

However, the results also showed that there were no differences between the dimensions of Attitudes and Concerns between participants who had enrolled in a special education course and those who did not. That is, one or two specialized courses in special education did not make a substantial difference in the participants’ attitudes toward inclusive education or alleviate their concerns about implementing inclusive education in their classroom. The possible explanation for this could be related to Chinese-specific special education policies of segregated special education schools (Qu, 2022; Wang et al., 2018) and the special education learning content pre-service general education teachers receive may be concentrated on special education within segregated schools and classrooms and not focused on the learning needs of future general education teachers who may be teaching in inclusive classrooms (Feng et al., 2016a).

The gap between research and practice in education, especially special education, has long been a critical issue (Martinez and Hallahan, 2000; McKenna et al., 2019; Klinger and Boardman, 2011). This also extends to the gap between the special education curriculum in teacher training programs and the skills teachers actually need in practice (McLeskey et al., 2018). Although some provinces and municipalities in China have integrated special education into general education courses, and district education bureaus emphasize its importance, research still shows a lack of special education training for general education teachers (Feng and Zhu, 2018; Sun, 2014). Many in-service and pre-service general education teachers in China report they have not received formal training for effective inclusive education (Ma and Tan, 2010; Zhu and Lei, 2014). In addition, this lack of training can differ by provinces. For example, general education teachers in the Eastern region of China show better understanding and a more favorable attitude toward inclusive education than general education teachers in the Western region of China (Sun, 2014). One reason could be because the Eastern region of China includes regions with higher economic revenue that can be utilized by the municipal governments as a means to fund better teacher training programs compared to the Western region that includes more rural areas and provinces with comparatively lower revenue.

The results of the current study showed a significant positive correlation between participants’ SCAIE-R and TEIP scores, suggesting a link between attitudes toward inclusive education and teacher self-efficacy. This aligns with previous research showing that teachers’ attitudes and beliefs are strongly connected to their teaching practices (Denessen et al., 2022; Wray et al., 2022). Teachers who feel confident in implementing inclusive education and collaborating with others are more effective in including students with disabilities (Cameron and Cook, 2013; Saloviita, 2020). Therefore, teacher training should emphasize behavior management for diverse classrooms, focusing on practical skills and opportunities in real-world settings (McLeskey et al., 2018; Yada and Savolainen, 2017).

In many universities in China and elsewhere, an ‘Introduction to Special Education’ course may be the only preparation pre-service general education teachers receive for inclusive education (Chan et al., 2023). The ‘Introduction to Special Education’ course typically includes a broad overview of the different types of disabilities under the Chinese education system (i.e., physical disabilities; neurological, intellectual, and developmental disabilities; vision-related disabilities; and hearing-related disabilities), how the disabilities are diagnosed, and some classroom management skills to teach students in an inclusive environment (Feng et al., 2016b). Teacher training programs need to offer as many special education courses as possible to pre-service general education teachers while also emphasizing the importance of acquiring pedagogical strategies and classroom behavioral management for diverse learners. These courses should be developed collaboratively by both academics and in-service teachers to bridge the gap between current training and actual classroom practice. As the demographic information of the current study showed, pre-service general education teachers have limited experience in special education or interactions with individuals with disabilities. Teacher training programs should also provide opportunities for pre-service general education students to learn in special education classroom field placements or participate in internships at schools with well-developed inclusive education practices.

### Limitations and recommendations for future research

First, the 587 participants in this study were from four colleges of education in Guangdong Province and thus may not represent all education students in China. Future studies should include students from various provinces and examine differences in teacher training programs and provincial policies. For example, a content analysis of teacher training curriculum from various teacher training institutions specific to inclusive education and special education may provide an overview of current teacher training practices and areas of improvement.

Second, the SCAIE-R and TEIP scales used in this study were originally in English. Despite a thorough translation process, the Chinese versions may not fully align with the English originals due to cultural and educational differences. This could have impacted the study’s internal validity. Our analysis found limited explanatory power of the TEIP scale, and the results of the SCAIE-R scale showed inconsistent results regarding the gender differences of participants. Future studies should consider using scales that may be better suited for Chinese participants or those that are validated for use in Chinese populations both in terms of language and culture.

Third, the study assumed uniformity in educational disability categories, overlooking the diverse needs within inclusive classrooms. For instance, students needing mobility assistance may have different requirements than those with learning disabilities. Even among students with the same category of disability, support needs can vary significantly. For example, autism spectrum disorder is one neurodevelopmental disability with a very heterogeneous population. Some students with autism spectrum disorder may not need support in academics but may need support in meaningful social interaction with peers, whereas some students with autism spectrum disorder may require extensive support in academics as well as social interaction and adaptive skills within general education classrooms. As individualized education is essential for effective inclusion, future research should target specific populations and the diversity within those populations to ensure teachers can be trained to support diverse students with evidence-based methods.

## Data Availability

The original contributions presented in the study are included in the article/Supplementary material, further inquiries can be directed to the corresponding author.
