# Supplementary material for: Chinese pre-service general education teachers’ attitudes and self-efficacy toward inclusive education
Source: Front Psychol. 2025 Sep 9;16:1490144. doi: 10.3389/fpsyg.2025.1490144 (PMC12454369; doi:10.3389/fpsyg.2025.1490144)
Supplement: Supplementary file 1 [file Supplementary_file_1.docx]

**Supplementary Materials**

**A. Items, Loading, and Reliability of the Sentiment, Attitudes, and Concerns about Inclusive Education-Revised**

| **Item** | **Sentiments** | **Attitude of acceptance** | **Concerns** |
| --- | --- | --- | --- |
| I find it difficult to overcome a sense of shock when I first encounter someone with a serious physical disability | 0.82 |  |  |
| I am afraid to look squarely at individuals with disability. | 0.89 |  |  |
| I tend to end contact with individuals with disabilities as quickly as possible. | 0.85 |  |  |
| I think that students who have difficulty expressing ideas in words should participate in the school’s regular curriculum |  | 0.78 |  |
| I believe students who often fail at exams should participate in the school’s regular curriculum. |  | 0.79 |  |
| I believe students who need individualized instruction should participate in the school’s regular curriculum. |  | 0.82 |  |
| I believe students who are often inattentive should participate in the school’s regular curriculum. |  | 0.84 |  |
| I believe students who use braille, sign language, etc. to communicate should participate in the school’s regular curriculum. |  | 0.80 |  |
| I am worried that my workload may increase due to the inclusion of students with disabilities in my class. |  |  | 0.72 |
| I am worried that I will not be able to pay enough attention to all the students in an inclusive classroom. |  |  | 0.83 |
| I am worried that my pressure may increase if there are students with disabilities in my class. |  |  | 0.75 |
| I am worried that students without disabilities may not accept students with disabilities. |  |  | 0.75 |
| I am worried that I lack the knowledge and skills needed to teach students with disabilities. |  |  | 0.64 |
| **Cronbach’s α by dimensions** | **0.83** | **0.87** | **0.80** |
| **Cronbach’s α of the questionnaire** | **0.73** | | |

**B. Items, Loading, and Reliability of the Teacher Efficacy for Inclusive Practices Scale**

| **Item** | **Inclusive instruction** | **Collaboration** | **Managing behavior** |
| --- | --- | --- | --- |
| I can use various assessment strategies such as adjustment to tests, practical evaluation, etc. | 0.78 |  |  |
| When students feel confused, I can provide them with alternative explanations or examples. | 0.76 |  |  |
| I believe that I can design learning tasks that reflect the individual needs of students with disabilities. | 0.74 |  |  |
| I can accurately evaluate how well students understand what I teach. | 0.77 |  |  |
| I can provide learners with tasks at a certain degree of difficulty. | 0.63 |  |  |
| I can collaborate with other professionals and staff (such as support staff, other teachers) to jointly support students with disabilities in class. |  | 0.65 |  |
| I believe that I have the ability to involve caregivers of students with disabilities in school activities. |  | 0.67 |  |
| I can make caregivers feel comfortable about coming to school. |  | 0.68 |  |
| I can collaborate with other experts (such as itinerate teachers, speech pathologists) to develop pedagogical plans for students with disabilities. |  | 0.81 |  |
| I believe that I can provide information on legal provisions and policies related to the inclusion of students with disabilities to individuals who are not familiar with them. |  | 0.76 |  |
| I can control problematic behaviors in the classroom. |  |  | 0.69 |
| I can calm down students who exhibit problematic behaviors or who are noisy in class. |  |  | 0.80 |
| I can bring my students in line with classroom rules. |  |  | 0.68 |
| I believe I can instruct students who are physically aggressive. |  |  | 0.83 |
| I can predict student behavior. |  |  | 0.71 |
| **Cronbach’s α by dimensions** | **0.88** | **0.88** | **0.86** |
| **Cronbach’s α of the questionnaire** | **0.93** |  | |
